# Supplementary material for: MAPL regulates gasdermin-mediated release of mtDNA from lysosomes to drive pyroptotic cell death
Source: Nat Cell Biol. 2025 Oct 13;27(10):1708–24. doi: 10.1038/s41556-025-01774-y (PMC12527936; doi:10.1038/s41556-025-01774-y)
Supplement: Supplementary file 1 — Supplementary Table and Video Figure Legends. Supplementary Table 3. Information of the primary and secondary antibodies and the dilutions used in immunoblots. Supplementary Table 4. Information of the primary and secondary antibodies and the dilutions used in immunofluorescent microscopy. Supplementary Table 5. Sequence of the primers used for PCR and qRT–PCR. [file 41556_2025_1774_MOESM1_ESM.pdf]

nature cell biology

Supplementary information https://doi.org/10.1038/s41556-025-01774-y

MAPL regulates gasdermin-mediated release of mtDNA from lysosomes to drive pyroptotic cell death

In the format provided by the authors and unedited

Supplementary Tables

Supplementary Table 3

Information of the primary and secondary antibodies and the dilutions used in immunoblots.

Supplementary Table 4

Information of the primary and secondary antibodies and the dilutions used in immunofluorescent microscopy.

Supplementary Table 5

Sequence of the primers used for PCR and qRT-PCR

Supplementary Videos

Supplementary Video 1:

Example of mtDNA (PicoGreen, green) release from the mitochondrial network (MitoTracker, magenta) in U2OS cells expressing MAPL for 24 hours. Still images are shown in Figure 3A.

Supplementary Video 2:

Example of mtDNA (PicoGreen, green) trafficking of cytosolic distance to a dextran-labelled lysosome (magenta) that already contains DNA in U2OSneo cells expressing MAPL for 24 hours. Mitochondria are also labelled in blue by MitoTracker. Still images are shown in Figure 4D.

Supplementary Video 3:

Example of mtDNA (PicoGreen, green) trafficking to a dextran-labelled lysosome (magenta) at the mitochondrial (MitoTracker, blue) surface U2OSneo cells expressing MAPL for 24 hours. Still images are shown in Figure 4E.

Supplementary Video 4:

Evidence that mtDNA (PicoGreen, green) can be released from GAL3-RFP-positive (magenta) lysosomes. This video was taken in U2OSRFP-GAL3 cells expressing MAPL for 48 hours. Mitochondria are also labelled with MitoTracker (blue). Still images are shown in Figure 6G.

Supplementary Table 3 Antibodies for Immunoblots

Primary antibody	Company	Catalogue Number/RRID	Dilution
Actin	Sigma	A2228/RRID:AB_476697	1:2000
AIF(E1)	Santa Cruz	Sc-13116/ RRID:AB_626654	1:1000
Caspase 1	Adipogen	AG-20B-0042-C100/	1:500
Cleaved caspase 3	Cell Signaling	96661S/RRID:AB_2341188	1:1000
Cleaved caspase 7	Cell Signaling	9491/ RRID:AB_2068144	1:1000
cGAS	Cell signaling	15102/RRID:AB_2732795	1:1000
FLAG	Sigma	F1804/RRID:AB_262044	1:1000
GSDMD	Abcam	ab210070 /RRID:AB_2893325	1:1000
GSDME	Abcam	ab215191/RRID:AB_2737000	1:1000
Histone 3	Abclonal	A2348/RRID:AB_2737000	1:1000
HA	Sigma	H9658/RRID:AB_260092	1:1000
IL1b	Bio-Techne	AB-401-NA/	1:1000
LAMP1	Cell Signaling	9091/RRID:AB_2687579	1:1000
LRRK2	Abcam	ab133474/RRID:AB_2713963	1:1000
MAPL	Sigma	HPA017681/RRID:AB_1848699	1:1000
MFN2(XX-1)	Santa Cruz	Sc-100560/RRID:AB_2235195	1:1000
NLRP3	Cell Signaling	15101/RRID:AB_2722591	1:1000
NF-kB p65	Abcam	ab16502/RRID:AB_443394	1:1000
PMP70	Abcam	ab3421/RRID:AB_2219901	1:2000
RHOT1	Sigma	HPA010687/RRID:AB_1079813	1:1000
p-STAT3	Abclonal	AP0474/RRID:AB_2771567	1:1000
TOM20	Sigma	HPA010687/RRID:AB_1080326	1:1000
UQCR2	Proteintech	83667-2/ RRID:AB_3671273	1:1000
Vinculin	Sigma	V4505/RRID:AB_477617	1:1000
VPS35	Abnova	H00055737/RRID:AB_566269	1:1000
**Secondary antibody (IF)**	**Company**	**Catalogue Number**	**Dilution**
HRP-anti-mouse	Cytiva	NA931/RRID:AB_772210	1:5000
HRP-anti-rabbit	Cytiva	NA934/RRID:AB_772206	1:5000

Supplementary Table 4 Antibodies for Immunoflourescence

Primary antibody (IF)	Company	Catalogue Number/RRID	Dilution
Complex I	Abcam	ab109798/ RRID:AB_10862214	1:500
dsDNA	Abcam	ab27156/RRID:AB_470907	1:1000
Galectin3-AF647	Biolegend	125408	1:1000
HA	Sigma	H9658/RRID:AB_260092	1:1000
LAMP1	Cell Signaling	9091/RRID:AB_260092	1:500
TOM40	Proteintech	18409-1-AP/RRID:AB_260092	1:1000
**Secondary antibody (IF)**	**Company**	**Catalogue Number/RRID**	**Dilution**
Mouse IgG Alexafluor 568	Invitrogen	A11031/ RRID:AB_144696	1:2000
Mouse IgG Alexafluor 488	Invitrogen	A11029/RRID:AB_2534088	1:2000
Mouse IgG2a Alexafluor 488	Invitrogen	A21131/ RRID:AB_2535771	1:2000
Mouse IgG2b Alexfluor 594	Invitrogen	A21145/RRID:AB_2535781	1:2000
Mouse IgG Alexafluor 647	Invitrogen	A21235/RRID:AB_2535804	1:2000
Rabbit IgG Alexafluor 647	Invitrogen	A21244/ RRID:AB_2535812	1:2000

Supplementary Table 5 Primers for PCR, qPCR and qRT-PCR

Gene name	Species	RefSeq ID	Forward sequence (5'-3')	Reverse sequence (5'-3')	Application
NLRP3	Human	NM_001127461.2	AAAGAGATGAGCCGAAGTGG	ATCCACTCCTCTTCAATGCTG	qRT-PCR
IL1B	Human	NM_000576.2	TACCTGTCCTGCGTGTTGAA	TCTTTGGGTAATTTTTGGGATCT	qRT-PCR
IL6	Human	NM_000600.3	GATGAGTACAAAAGTCCTGATCCA	CTGCAGCCACTGGTTCTGT	qRT-PCR
IFNA4	Human	NM_021068	AACTCCTCCTCGGGGAATC	AACTCCTCCTCGGGGAATC	qRT-PCR
IFNB1	Human	NM_002176.2	CGACACTGTTCGTGTTGTCA	GAAGCACAACAGGAGAGCAA	qRT-PCR
PPIA	Human	NM_021130	CCTAAAGCATACGGGTCCTG	TTTCACTTTGCCAAACACCA	qRT-PCR
ND1	Human	NC_012920.1 3307-4262	CCACCCTTATCACAACACAAGA	TCATATTATGGCCAAGGGTCA	qRT-PCR
ND1	Human	NC_012920.1 3307-4262	TAAAACCCGCCACATCTACCATCA	GAGTTCAGGGGAGAGTGCGTCATA	PCR
16S (or RNR2)	Human	NC_012920.1 1671-3229	TACCGCAAGGGAAAGATGAAAAAT	AGGCTTATGCGGAGGAGAATGTT	PCR
CO1	Human	NC_012920.1:5904-7445	CGTTTCCCCGCATAAACAACA	CCGGCCTCCACTATAGCAGA	qPCR
ND6	Human	NC_012920.1 14148-14672	ATTGGTGCTGTGGGTGAAA	CCTGACCCCTCTCCTTCATA	qPCR
TERT	Human	NC_000005.10	GAGGCCAGAGCAGTGAACA	CAGACACTTCTCCCCATTGC	qPCR
