# Supplementary material for: MAPL regulates gasdermin-mediated release of mtDNA from lysosomes to drive pyroptotic cell death
Source: Nat Cell Biol. 2025 Oct 13;27(10):1708–24. doi: 10.1038/s41556-025-01774-y (PMC12527936; doi:10.1038/s41556-025-01774-y)
Supplement: Supplementary file 2 — Reporting Summary [file 41556_2025_1774_MOESM2_ESM.pdf]

nature portfolio

Corresponding author(s): Heidi M. McBride

Last updated by author(s): 2025 07 04

Reporting Summary

Nature Portfolio wishes to improve the reproducibility of the work that we publish. This form provides structure for consistency and transparency in reporting. For further information on Nature Portfolio policies, see our Editorial Policies and the Editorial Policy Checklist.

Statistics

For all statistical analyses, confirm that the following items are present in the figure legend, table legend, main text, or Methods section.

n/a	Confirmed	
☐	☒	The exact sample size (*n*) for each experimental group/condition, given as a discrete number and unit of measurement
☐	☒	A statement on whether measurements were taken from distinct samples or whether the same sample was measured repeatedly
☐	☒	The statistical test(s) used AND whether they are one- or two-sided<br>*Only common tests should be described solely by name; describe more complex techniques in the Methods section.*
☐	☒	A description of all covariates tested
☐	☒	A description of any assumptions or corrections, such as tests of normality and adjustment for multiple comparisons
☐	☒	A full description of the statistical parameters including central tendency (e.g. means) or other basic estimates (e.g. regression coefficient) AND variation (e.g. standard deviation) or associated estimates of uncertainty (e.g. confidence intervals)
☐	☒	For null hypothesis testing, the test statistic (e.g. *F*, *t*, *r*) with confidence intervals, effect sizes, degrees of freedom and *P* value noted<br>*Give P values as exact values whenever suitable.*
☒	☐	For Bayesian analysis, information on the choice of priors and Markov chain Monte Carlo settings
☒	☐	For hierarchical and complex designs, identification of the appropriate level for tests and full reporting of outcomes
☒	☐	Estimates of effect sizes (e.g. Cohen's *d*, Pearson's *r*), indicating how they were calculated

Our web collection on statistics for biologists contains articles on many of the points above.

Software and code

Policy information about availability of computer code

Data collection	Methods outline all softwares used with relevant citations. Confocal images and analysis was done with MetaMorph software (MetaMorph Microscopy Automation and Image Analysis Software (RRID:SCR_002368). RNAseq reads were mapped using HISAT2 to reference mouse genome build mm10, and counted using featureCounts. Differential expression analysis was done using DeSeq2. Heatmaps were generated using ComplexHeatmaps, PCA plot values were computed in R and plotted using Prism9, and scatter dot plots were also plotted using Prism 9. For visualisation of read alignment, BAM indexes were generated using using SAMtools, and visualised using WashU Epigenome Browser.
Data analysis	Images were processed using FIJI Image J software (ImageJ (RRID:SCR_003070). For the genome-wide screen, sequencing reads were mapped to the library using xcalibr (https://www.thermofisher.com/order/catalog/product/OPTON-30965) and counts were then analyzed with MAGeCK (https://sourceforge.net/p/mageck/wiki/Home/, version 0.5.8) using the Robust Rank Aggregation (RRA) algorithm to identify genes whose perturbation (knockout or overexpression) primarily enhanced fitness in the MAPL overexpressing group but not the control group. For RNAseq, calculations were computed using Rstudio (RRID:SCR_000432, https://posit.co/, version 2021.09.1), and graphs plotted using Prism10 (GraphPad Prism, RRID:SCR_002798, http://www.graphpad.com/)

For manuscripts utilizing custom algorithms or software that are central to the research but not yet described in published literature, software must be made available to editors and reviewers. We strongly encourage code deposition in a community repository (e.g. GitHub). See the Nature Portfolio guidelines for submitting code & software for further information.

Data

Policy information about availability of data

All manuscripts must include a data availability statement. This statement should provide the following information, where applicable:

- Accession codes, unique identifiers, or web links for publicly available datasets
- A description of any restrictions on data availability
- For clinical datasets or third party data, please ensure that the statement adheres to our policy

Data availability statement is included in manuscript. RNAseq datasets are uploaded to GEO repository, and has been released. https://www.ncbi.nlm.nih.gov/geo/query/acc.cgi?acc=GSE301127. Data and materials are also deposited at https://zenodo.org/records/16387131 and has been released to the public

Research involving human participants, their data, or biological material

Policy information about studies with human participants or human data. See also policy information about sex, gender (identity/presentation), and sexual orientation and race, ethnicity and racism.

Reporting on sex and gender	Gender is not relevant. Manuscript is primarily using cultured cell lines that include both male and female lines (U2OS - female, BMDM were taken from both male and female mice.
Reporting on race, ethnicity, or other socially relevant groupings	N/A
Population characteristics	N/A
Recruitment	N/A
Ethics oversight	McGill Animal Care protocols approved for mouse colonies used in the generation of primary BMDM.

Note that full information on the approval of the study protocol must also be provided in the manuscript.

Field-specific reporting

Please select the one below that is the best fit for your research. If you are not sure, read the appropriate sections before making your selection.

☒ Life sciences ☐ Behavioural & social sciences ☐ Ecological, evolutionary & environmental sciences

For a reference copy of the document with all sections, see nature.com/documents/nr-reporting-summary-flat.pdf

Life sciences study design

All studies must disclose on these points even when the disclosure is negative.

Sample size	No statistical method was used to predetermine sample size. At least three independent experiments were performed to test for statistical significant differences. Minimum of 30 cells imaged per condition, in triplicate with at least 3 biological replicates. The design achieved sufficient size for statistical analysis as reported in previous publications within the field.
Data exclusions	No experimental data points were excluded. Only data from experiments with appropriate negative/positive controls is included in this study.
Replication	At least three independent experiments were performed to test for statistical significance and verify reproducibility of the experiments. We can confirm that all attempts at replication were successful. For videos, microscopy images and western blots only representative data is shown.
Randomization	No randomization was performed in this study. The experimental set-up included clearly defined groups/ conditions for comparison. To avoid human bias, automated software with identical settings between conditions was used for quantifications. Image analysis was performed randomized and blinded.
Blinding	Not necessary for most parts of this study due to automated quantification by a software with identical parameter settings. Image analysis was performed randomized and blinded.

Reporting for specific materials, systems and methods

We require information from authors about some types of materials, experimental systems and methods used in many studies. Here, indicate whether each material, system or method listed is relevant to your study. If you are not sure if a list item applies to your research, read the appropriate section before selecting a response.

Materials & experimental systems

n/a	Involved in the study	
☐	☒	Antibodies
☐	☒	Eukaryotic cell lines
☒	☐	Palaeontology and archaeology
☐	☒	Animals and other organisms
☒	☐	Clinical data
☒	☐	Dual use research of concern
☒	☐	Plants

Methods

n/a	Involved in the study	
☒	☐	ChIP-seq
☒	☐	Flow cytometry
☒	☐	MRI-based neuroimaging

Antibodies

Antibodies used

Primary antibody Company Catalogue Number/RRID Dilution
Actin Sigma A2228/RRID:AB_476697 1:2000
AIF(E1) Santa Cruz Sc-13116/ RRID:AB_626654 1:1000
Caspase 1 Adipogen AG-20B-0042-C100/ 1:500
Cleaved caspase 3 Cell Signaling 96661S/RRID:AB_2341188 1:1000
Cleaved caspase 7 Cell Signaling 9491/ RRID:AB_2068144 1:1000
cGAS Cell signaling 15102/RRID:AB_2732795 1:1000
FLAG Sigma F1804/RRID:AB_262044 1:1000
GSDMD Abcam ab210070 /RRID:AB_2893325 1:1000
GSDME Abcam ab215191/RRID:AB_2737000 1:1000
Histone 3 Abclonal A2348/RRID:AB_2737000 1:1000
HA Sigma H9658/RRID:AB_260092 1:1000
IL1b Bio-Techne AB-401-NA/ 1:1000
LAMP1 Cell Signaling 9091/RRID:AB_2687579 1:1000
LRRK2 Abcam ab133474/RRID:AB_2713963 1:1000
MAPL Sigma HPA017681/RRID:AB_1848699 1:1000
MFN2(XX-1) Santa Cruz Sc-100560/RRID:AB_2235195 1:1000
NLRP3 Cell Signaling 15101/RRID:AB_2722591 1:1000
NF-kB p65 Abcam ab16502/RRID:AB_443394 1:1000
PMP70 Abcam ab3421/RRID:AB_2219901 1:2000
RHOT1 Sigma HPA010687/RRID:AB_1079813 1:1000
p-STAT3 Abclonal AP0474/RRID:AB_2771567 1:1000
TOM20 Sigma HPA010687/RRID:AB_1080326 1:1000
UQCR2 Proteintech 83667-2/ RRID:AB_3671273 1:1000
Vinculin Sigma V4505/RRID:AB_477617 1:1000
VPS35 Abnova H00055737/RRID:AB_566269 1:1000

Secondary antibody (IF) Company Catalogue Number Dilution
HRP-anti-mouse Cytiva NA931/RRID:AB_772210 1:5000
HRP-anti-rabbit Cytiva NA934/RRID:AB_772206 1:5000

Primary antibody Company Catalogue Number/RRID Dilution
Actin Sigma A2228/RRID:AB_476697 1:2000
AIF(E1) Santa Cruz Sc-13116/ RRID:AB_626654 1:1000
Caspase 1 Adipogen AG-20B-0042-C100/ 1:500
Cleaved caspase 3 Cell Signaling 96661S/RRID:AB_2341188 1:1000
Cleaved caspase 7 Cell Signaling 9491/ RRID:AB_2068144 1:1000
cGAS Cell signaling 15102/RRID:AB_2732795 1:1000
FLAG Sigma F1804/RRID:AB_262044 1:1000
GSDMD Abcam ab210070 /RRID:AB_2893325 1:1000
GSDME Abcam ab215191/RRID:AB_2737000 1:1000
Histone 3 Abclonal A2348/RRID:AB_2737000 1:1000
HA Sigma H9658/RRID:AB_260092 1:1000
IL1b Bio-Techne AB-401-NA/ 1:1000
LAMP1 Cell Signaling 9091/RRID:AB_2687579 1:1000
LRRK2 Abcam ab133474/RRID:AB_2713963 1:1000
MAPL Sigma HPA017681/RRID:AB_1848699 1:1000
MFN2(XX-1) Santa Cruz Sc-100560/RRID:AB_2235195 1:1000
NLRP3 Cell Signaling 15101/RRID:AB_2722591 1:1000
NF-kB p65 Abcam ab16502/RRID:AB_443394 1:1000
PMP70 Abcam ab3421/RRID:AB_2219901 1:2000
RHOT1 Sigma HPA010687/RRID:AB_1079813 1:1000
p-STAT3 Abclonal AP0474/RRID:AB_2771567 1:1000
TOM20 Sigma HPA010687/RRID:AB_1080326 1:1000
UQCR2 Proteintech 83667-2/ RRID:AB_3671273 1:1000
Vinculin Sigma V4505/RRID:AB_477617 1:1000
VPS35 Abnova H00055737/RRID:AB_566269 1:1000

	Secondary antibody (IF) Company Catalogue Number Dilution<br>HRP-anti-mouse Cytiva NA931/RRID:AB_772210 1:5000<br>HRP-anti-rabbit Cytiva NA934/RRID:AB_772206 1:5000
Validation	Many of the antibodies were validated in our study using silencing or knockout lines, including MAPL, LRRK2, VPS35, VDAC1, MIRO1, MIRO2, GSDMD, GSDME, cGAS. Other antibodies are well established, company validated on their websites, and routinely published

Eukaryotic cell lines

Policy information about cell lines and Sex and Gender in Research

Cell line source(s)	U2OS cells (RRID:CVCL_0042)<br>TMEM192-3xHA (U2OSTMEM192-3xHA) (RRID:Addgene 102930)<br>pmRFP-C1-Galectin-3 (U2OSRFP-Gal3)<br>pcDNA3.1(U2OSneo) (Thermo Fisher, V79020)<br>HUH-7 (RRID:CVCL_0336),<br>143B Human osteosarcoma cell line (ATCC CRL-8303, gift from E. Shoubridge, McGill University)<br>BMK and BMK Bax/Bak-/- (Eileen White, see PMID: 12242152)
Authentication	No additional authentication was performed.
Mycoplasma contamination	All cell lines were frequently tested for mycoplasma contamination. No mycoplasma contamination was detected during the duration of this study.
Commonly misidentified lines<br>(See ICLAC register)	No ICLAC registered commonly misidentified cell line was used in this study.

Animals and other research organisms

Policy information about studies involving animals; ARRIVE guidelines recommended for reporting animal research, and Sex and Gender in Research

Laboratory animals	MAPL KO on C57BL/6J background, registered as RRID:MGI:7524577<br>LRRK2 KO on C57BL/6J background, registered as .RRID:IMSR_JAX:012444
Wild animals	No wild animals used. All breedings done from heterozygous animals so wild type mice came from littermate controls
Reporting on sex	BMDM were isolated from mixed male and female. Experiments on male or female BMDM individually did not change any of the results across the replicates. The manuscript will report exactly which replicates are from which sex BMDM, and when they are pooled together.
Field-collected samples	N/A
Ethics oversight	Animal experimentation was conducted in accordance with the guidelines of the Canadian Council for Animal Care. Protocols were approved by the Animal Care Committees of McGill University.

Note that full information on the approval of the study protocol must also be provided in the manuscript.

Plants

Seed stocks	N/A
Novel plant genotypes	N/A
Authentication	N/A
