# Supplementary material for: MAPL regulates gasdermin-mediated release of mtDNA from lysosomes to drive pyroptotic cell death
Source: Nat Cell Biol. 2025 Oct 13;27(10):1708–24. doi: 10.1038/s41556-025-01774-y (PMC12527936; doi:10.1038/s41556-025-01774-y)

Figure 1A

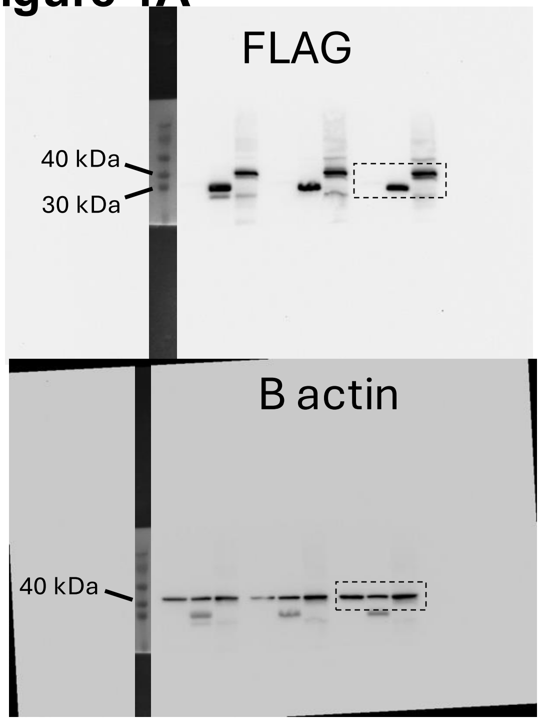

Figure 1J

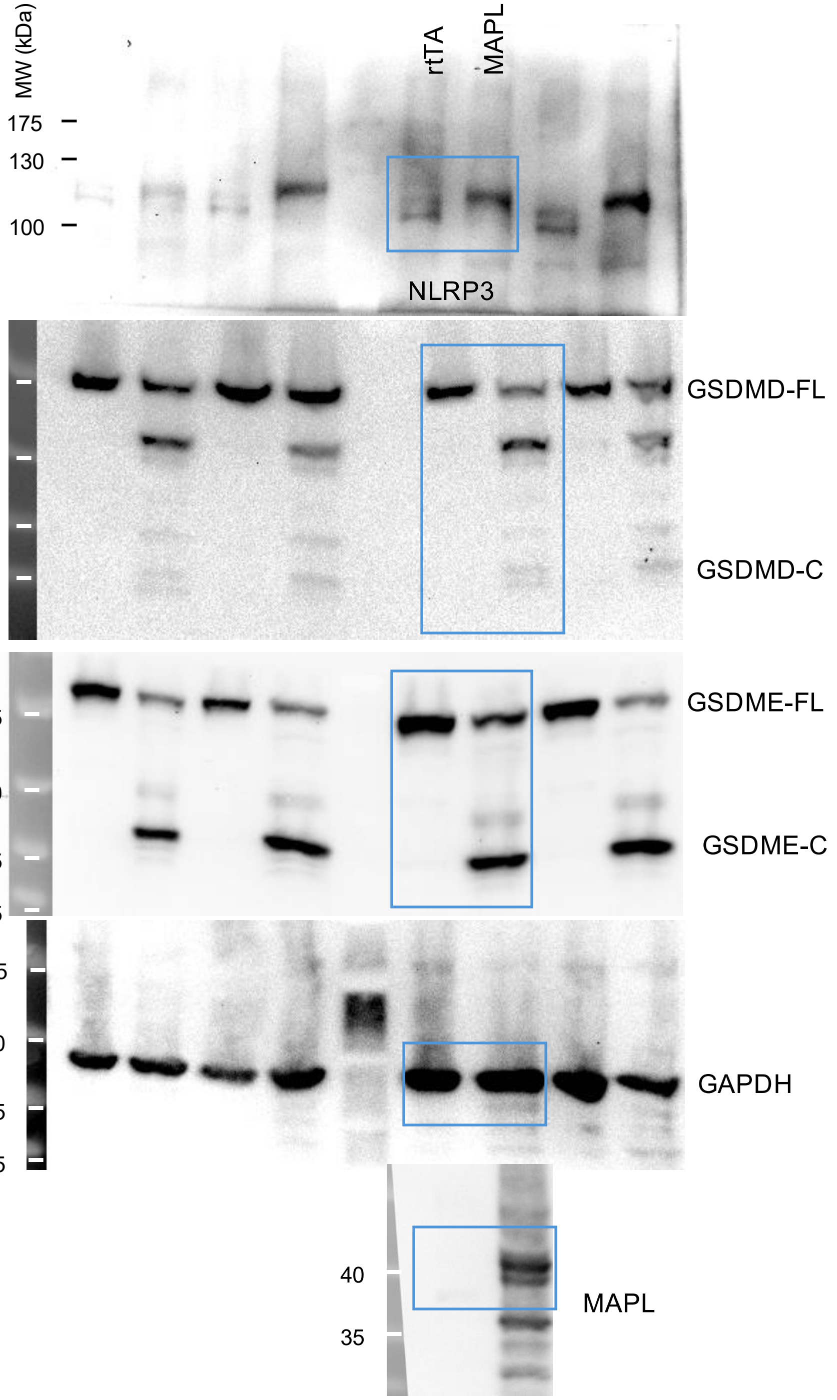

Figure 1C

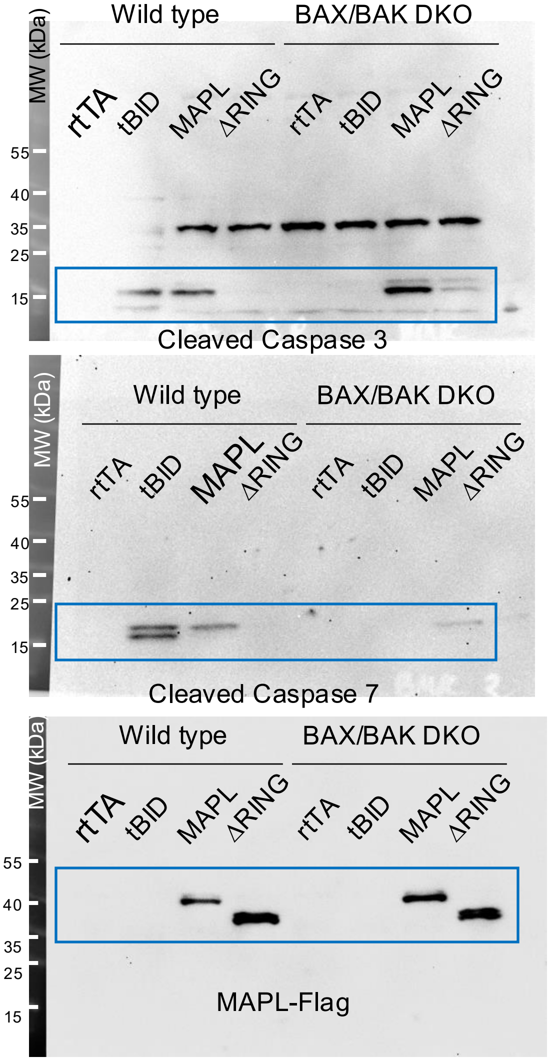

Figure 2E

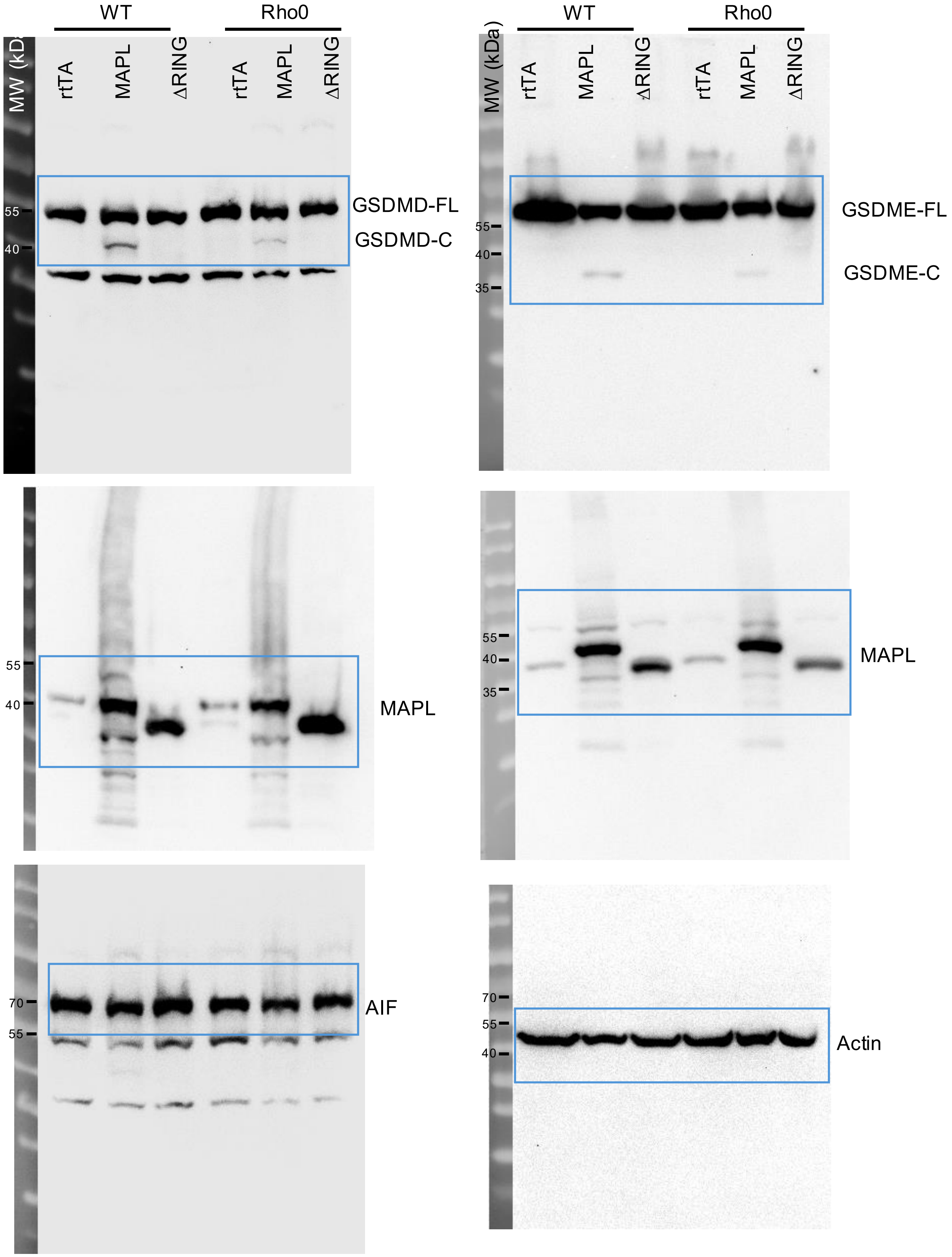

WT
Rho0
MW (kDa)
rtTA
MAPL
ΔRING
rtTA
MAPL
ΔRING
55
40
GSDMD-FL
GSDMD-C
WT
Rho0
MW (kDa)
rtTA
MAPL
ΔRING
rtTA
MAPL
ΔRING
55
40
35
GSDME-FL
GSDME-C
55
40
MAPL
55
40
35
MAPL
70
55
AIF
70
55
40
Actin

Figure 2K

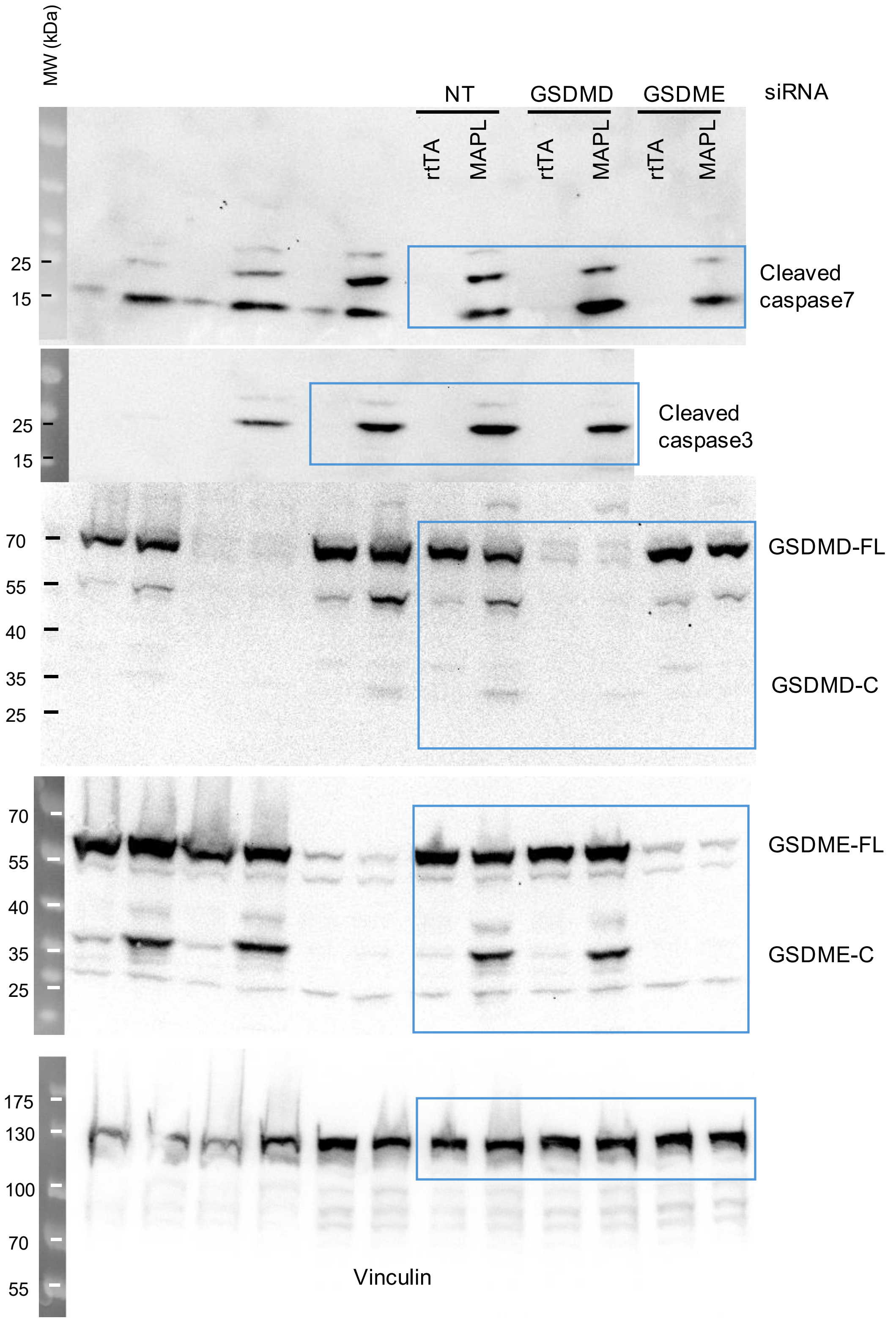

MW (kDa)
NT
GSDMD
GSDME
siRNA
rtTA
MAPL
rtTA
MAPL
rtTA
MAPL
25
15
Cleaved caspase7
25
15
Cleaved caspase3
70
55
40
35
25
GSDMD-FL
GSDMD-C
70
55
40
35
25
GSDME-FL
GSDME-C
175
130
100
70
55
Vinculin

Figure 6L

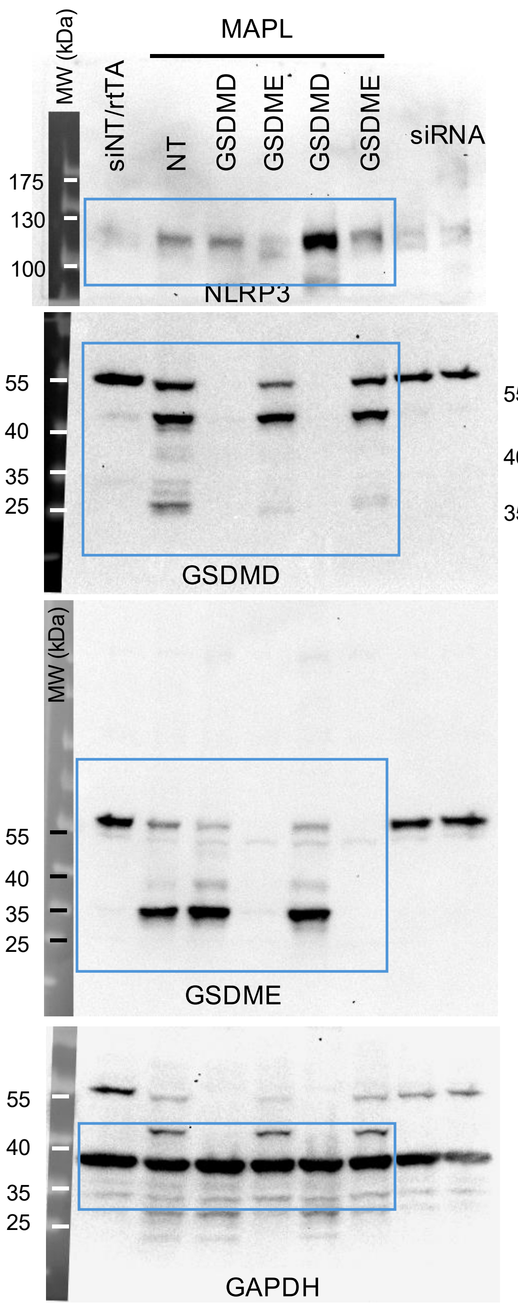

Figure 6M

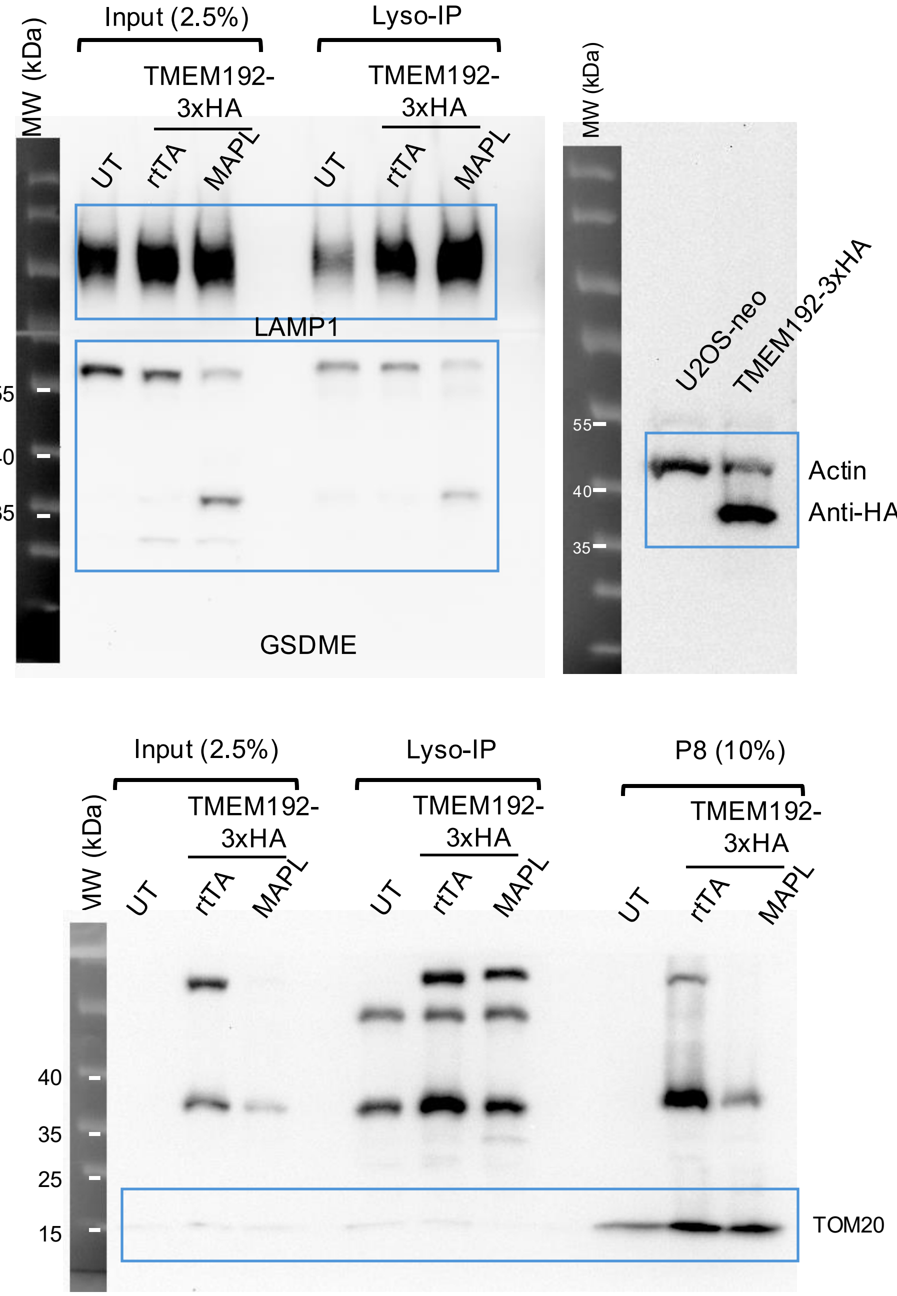

Figure 7A

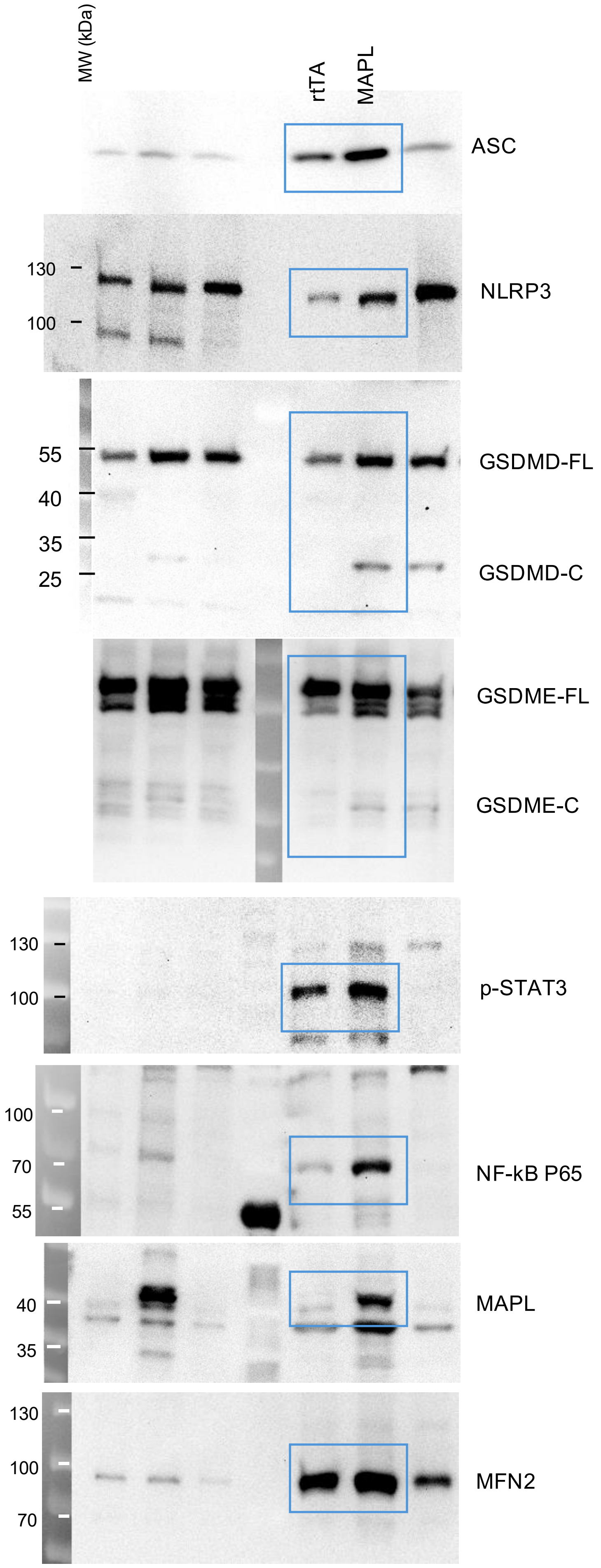

MW (kDa)
rtTA
MAPL
ASC
130
100
NLRP3
55
40
35
25
GSDMD-FL
GSDMD-C
GSDME-FL
GSDME-C
130
100
p-STAT3
100
70
55
NF-kB P65
40
35
MAPL
130
100
70
MFN2

Figure 7G

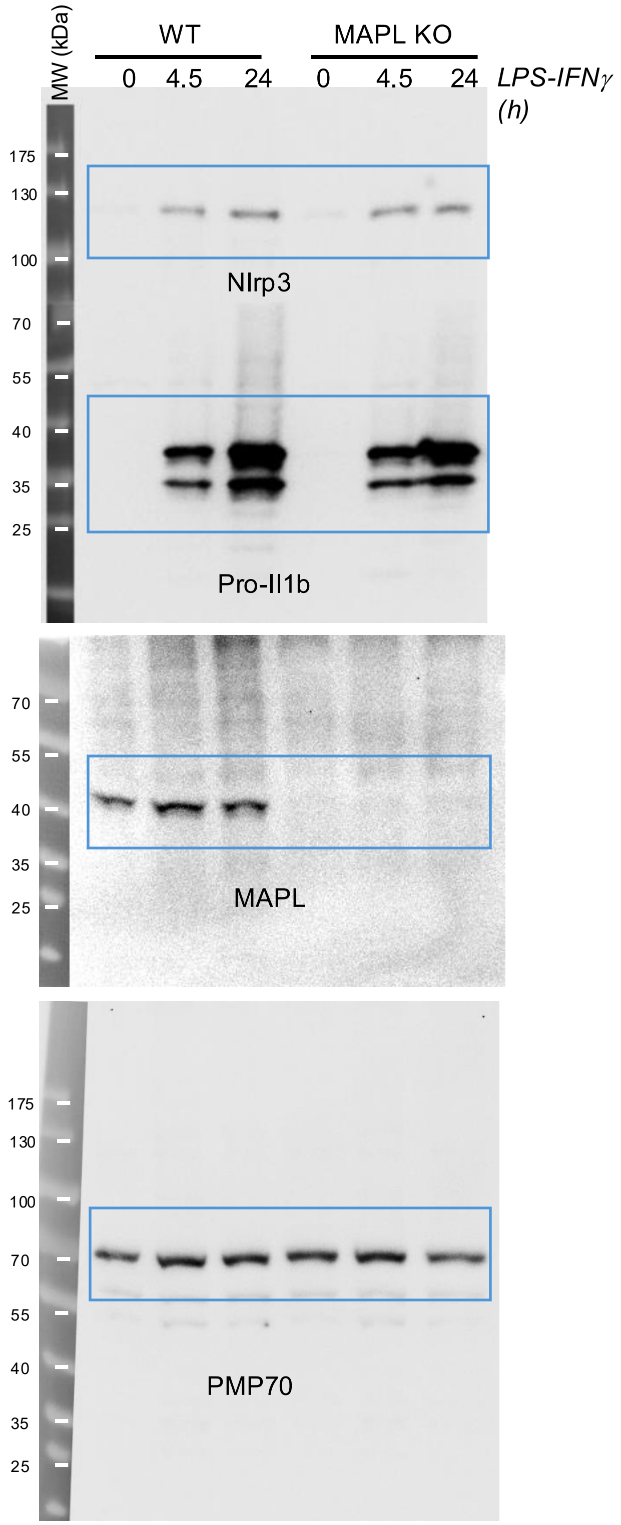

Figure 7K

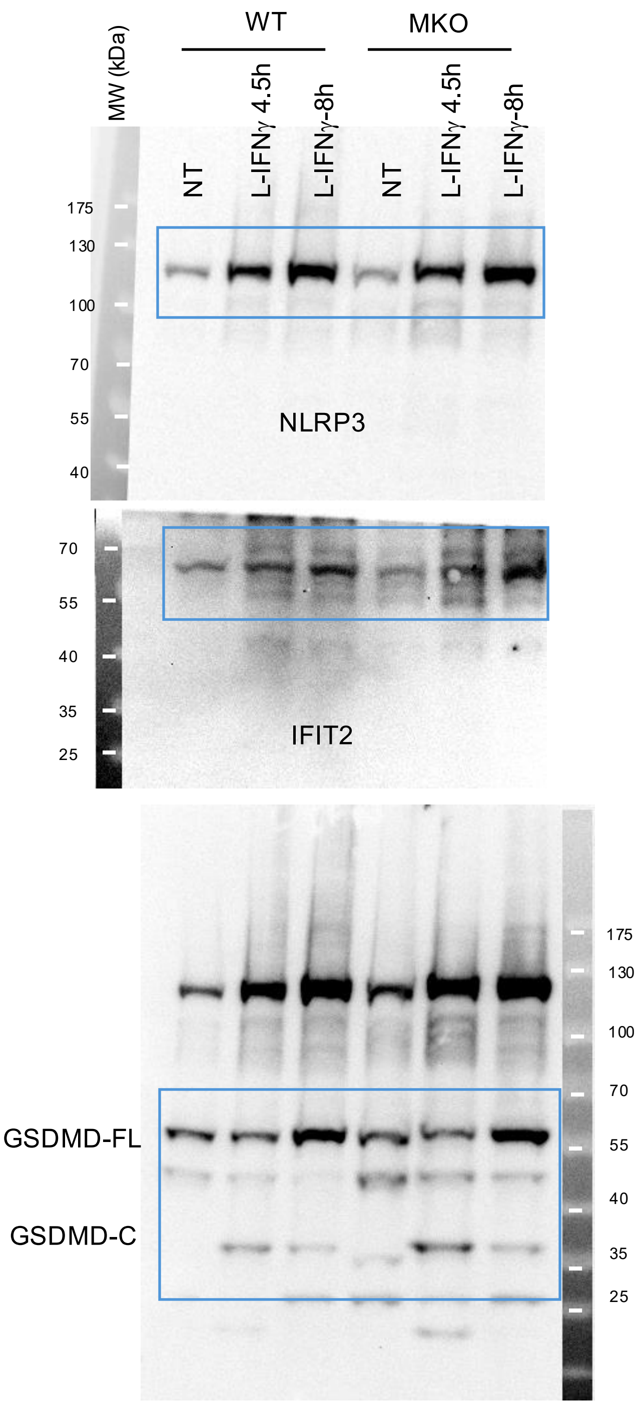

Extended Data Fig 1B

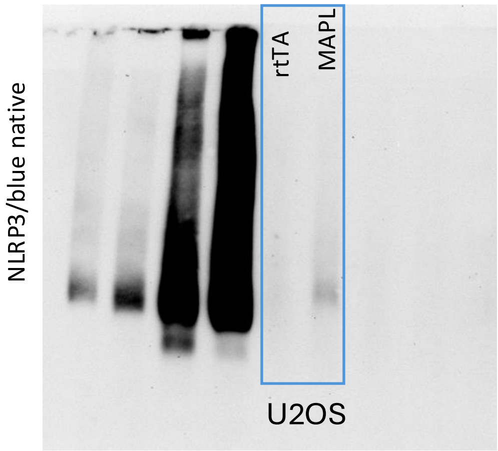

Extended Data Fig. 1E

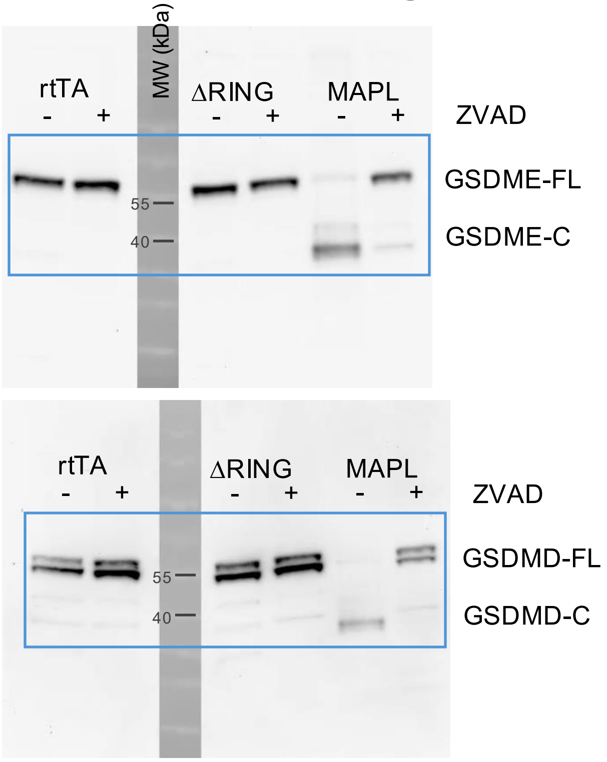

Extended Data Fig 2A

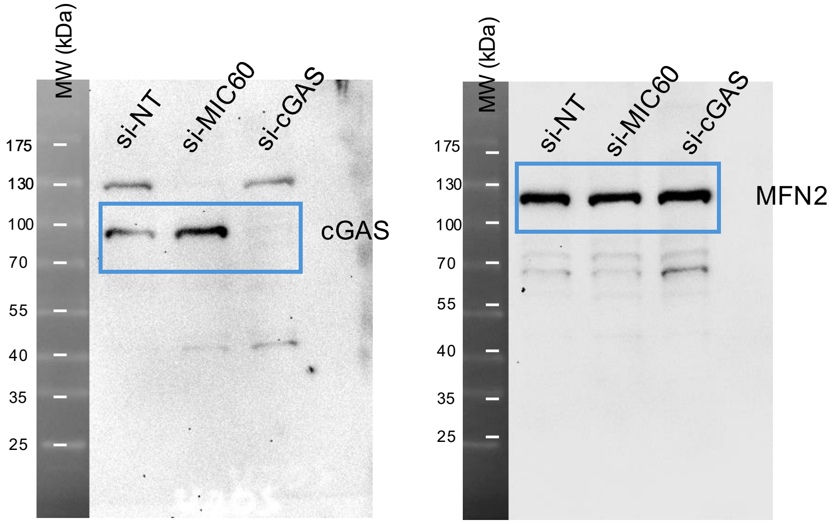

Extended Data Fig 3A

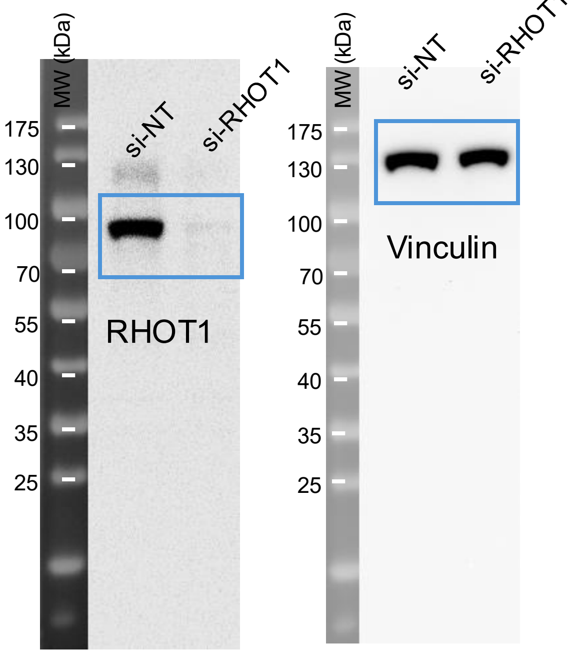

Extended Data Fig 3C

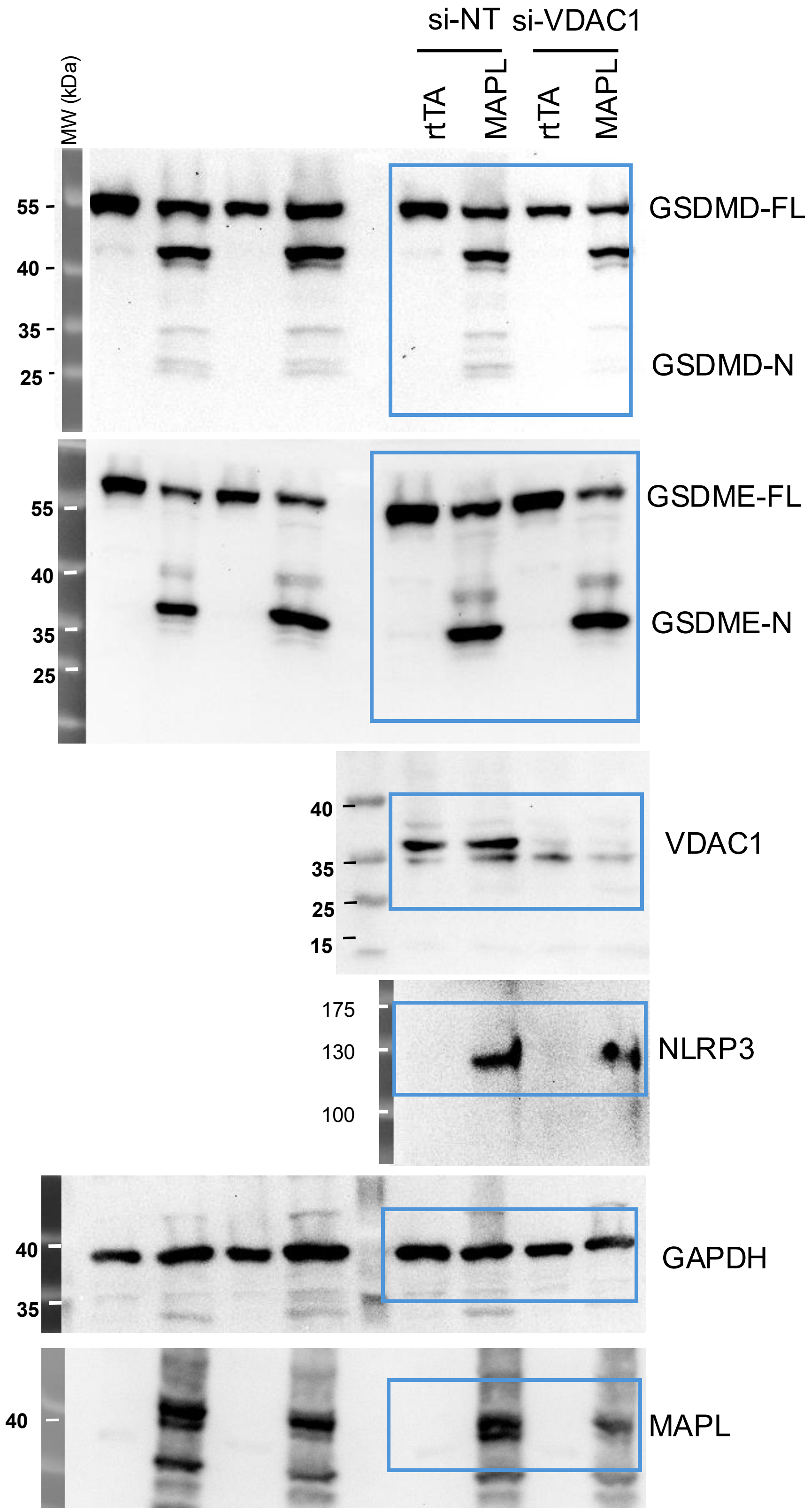

Extended Data Fig 5A

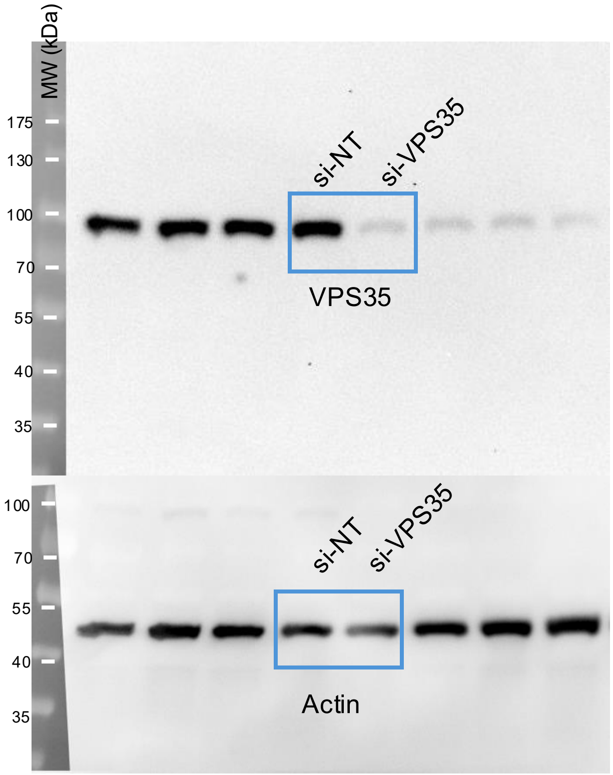

Extended Data Fig 5B

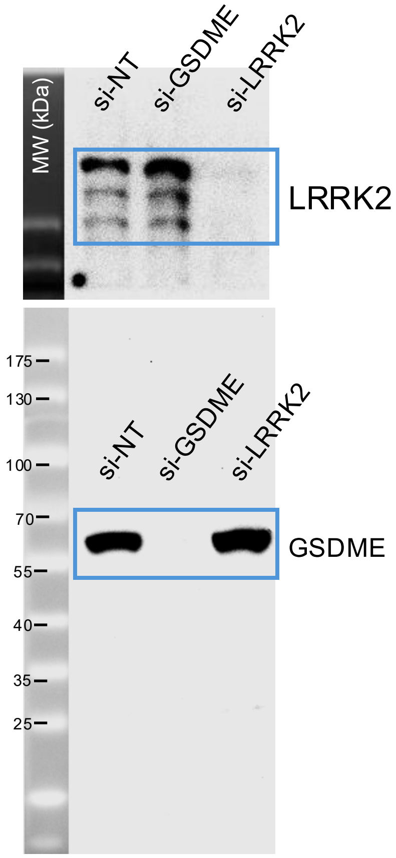

Extended Data Fig 6

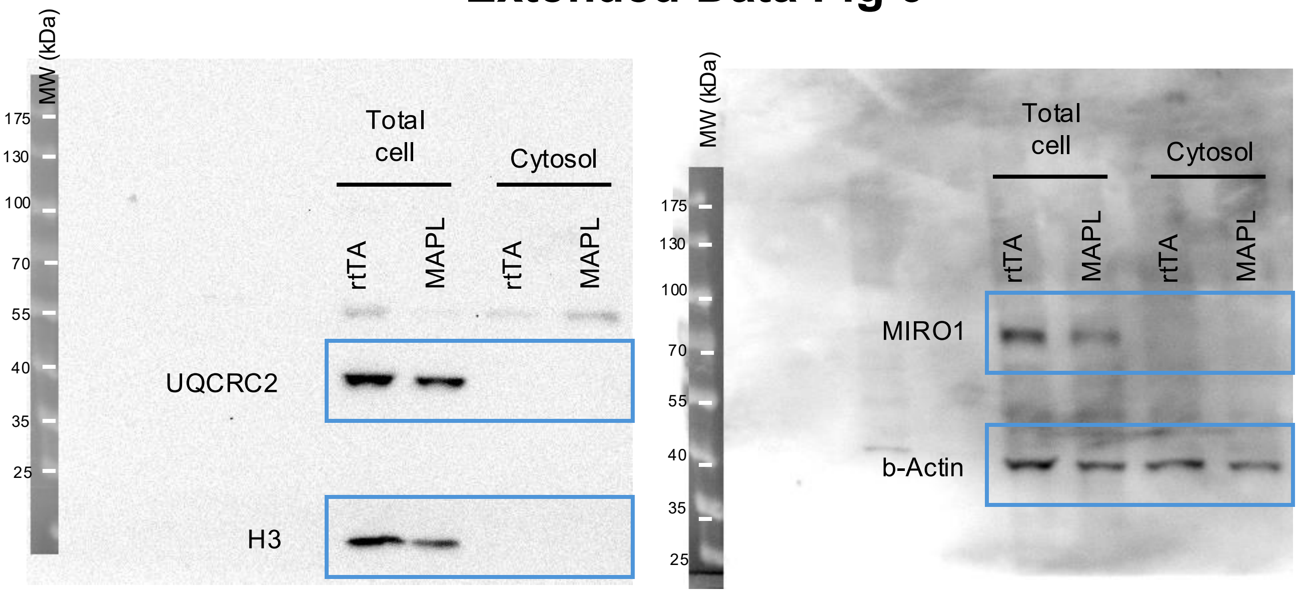

Extended Data Fig 7A

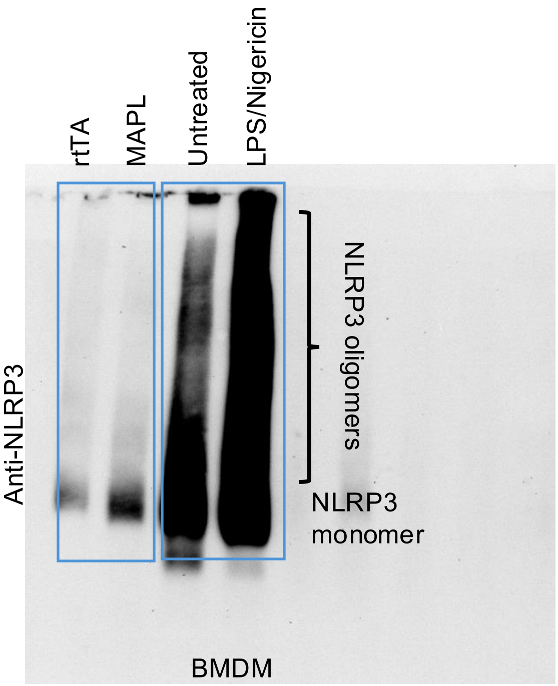

Extended Data Fig 7B

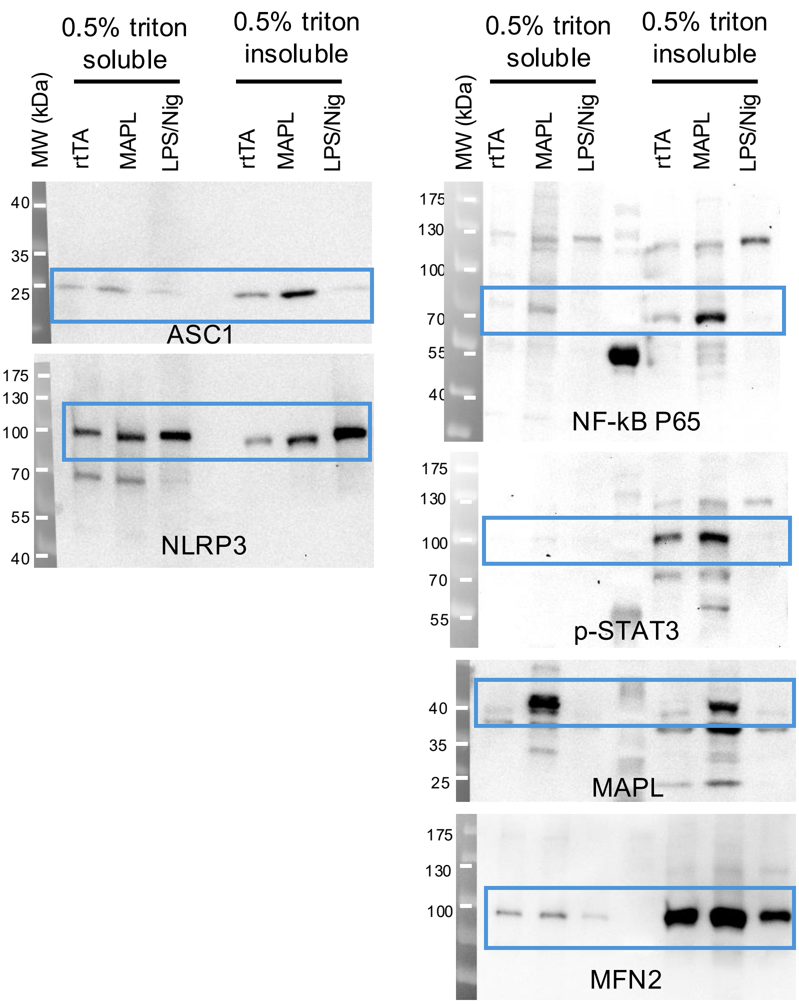

Extended Data Fig 7C

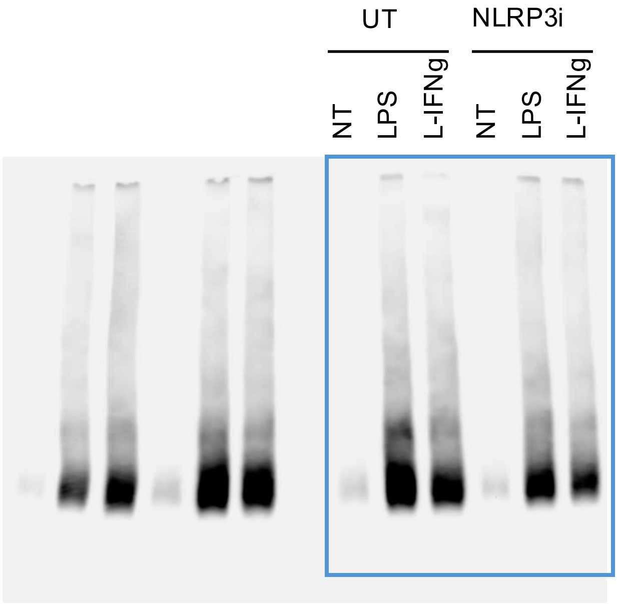

Extended Data Fig 7D

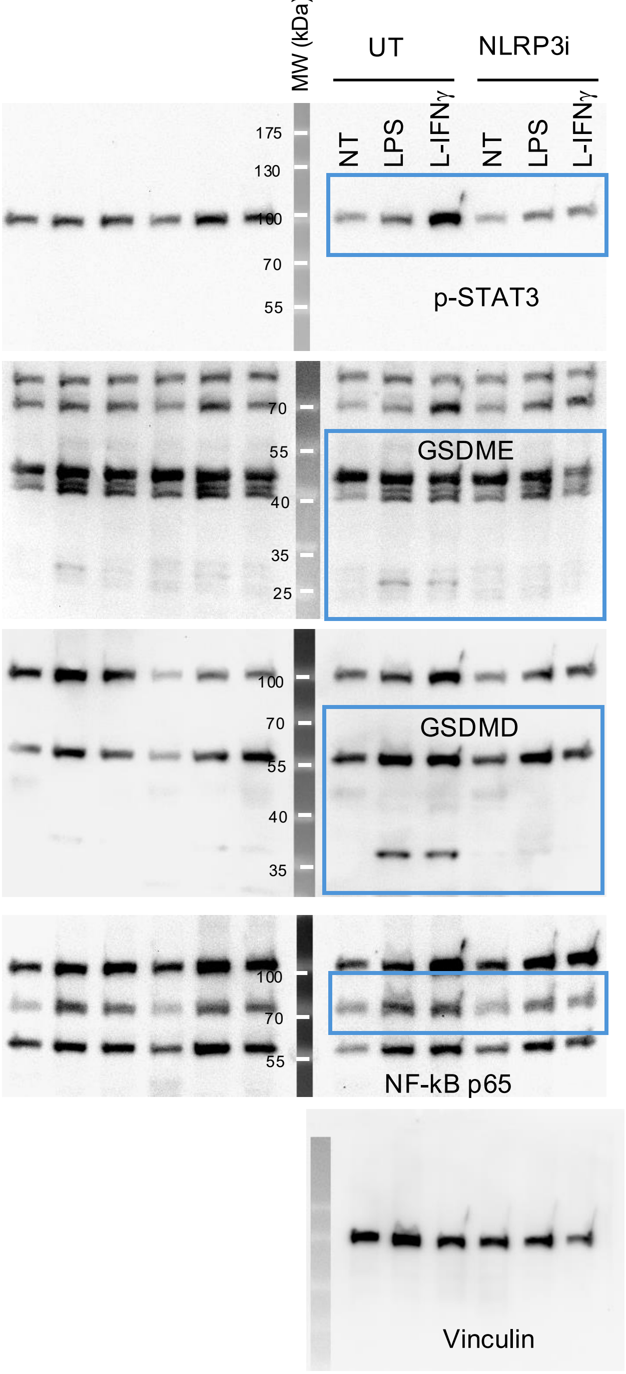

Extended Data Fig 7E

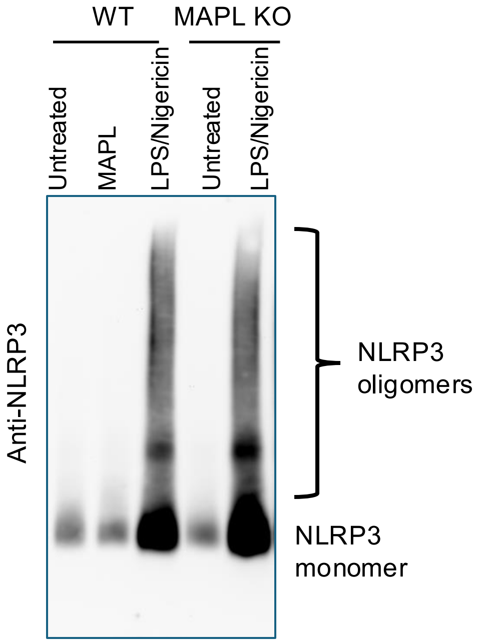

Extended Data Fig 7F

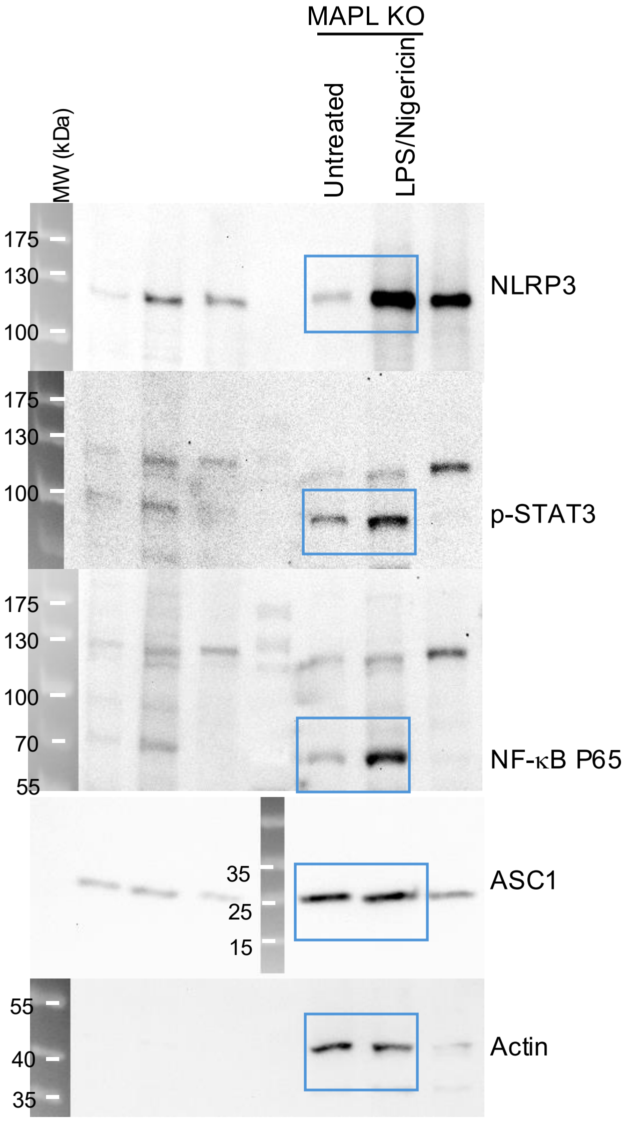

Supplement: Supplementary file 10 — Single file with labelled uncropped blots for each figure and extended data figure. [file 41556_2025_1774_MOESM10_ESM.pdf]
